# Vocal Fold Augmentation with Injectable Polycaprolactone Microspheres/Pluronic F127 Hydrogel: Long-Term *In Vivo* Study for the Treatment of Glottal Insufficiency

**DOI:** 10.1371/journal.pone.0085512

**Published:** 2014-01-22

**Authors:** Seong Keun Kwon, Hee-Bok Kim, Jae-Jun Song, Chang Gun Cho, Seok-Won Park, Jong-Sun Choi, Junsun Ryu, Se Heang Oh, Jin Ho Lee

**Affiliations:** 1 Department of Otorhinolaryngology, Head and Neck Surgery, Seoul National University Hospital, Seoul, Republic of Korea; 2 Cancer Research Institute, Seoul, Republic of Korea; 3 Seoul National University Medical Research Center, Seoul, Republic of Korea; 4 Department of Otorhinolaryngology, Head and Neck Surgery, Dongguk University Ilsan Hospital, Goyang, Republic of Korea; 5 Department of Pathology, Dongguk University Ilsan Hospital, Goyang, Republic of Korea; 6 Head and Neck Oncology Clinic, National Cancer Center, Goyang, Republic of Korea; 7 Department of Nanobiomedical Science & WCU Research Center, Dankook University, Cheonan, Republic of Korea; 8 Department of Advanced Materials, Hannam University, Yuseong Gu, Daejeon, Republic of Korea; University of Akron, United States of America

## Abstract

There is increasing demand for reconstruction of glottal insufficiency. Several injection materials have been examined for this purpose, but all had limitations, such as poor long-term durability, migration from the injection site, inflammation, granuloma formation, and interference with vocal fold vibration due to viscoelastic mismatch. Here, we developed a novel injection material, consisting of polycaprolactone (PCL) microspheres, which exhibits better viscoelasticity than conventional materials, and Pluronic F127 carrier, which decreases the migration of the injection materials. The material was injected into rabbits with glottal insufficiency and compared with the FDA-approved injection material, calcium hydroxylapatite (CaHA). Endoscopic and histological examinations indicated that PCL/Pluronic F127 remained at the injection site with no inflammatory response or granuloma formation, whereas CaHA leaked out and migrated from the injection site. Therefore, vocal fold augmentation was almost completely retained during the 12-month follow-up period in this study. Moreover, induced phonation and high-speed recording of vocal fold vibration showed decreased vocal fold gap area in the PCL/Pluronic F127 group. Our newly developed injection material, PCL/Pluronic F127, permits efficient augmentation of paralyzed vocal fold without complications, a concept that can be applied clinically, as demonstrated by the successful long-term follow-up.

## Introduction

The main functions of the larynx are to protect the lower airways and to produce the voice, which are accomplished by contact and closure of the two vocal folds. Glottal insufficiency due to incomplete contact between the two vocal folds may be caused by several glottal conditions such as vocal fold palsy or paresis, presbylaryngis, scarring, and sulcus vocalis. [1] Injection laryngoplasty has been used to enhance vocal fold contact and treat many types of glottal insufficiency. Many different materials have been developed for vocal fold injection. [2] Bruening performed the first injection augmentation of the vocal folds using paraffin in 1911. [3] Since then, several injectable substances have been used to treat glottal insufficiency, including fat, [4] fascia, [5] gel foam, [6] collagen, [7] silicone, [8] Teflon, [9] and others. [10] Each of these substances has demonstrated limitations such as poor long-term durability, migration from the injection site, inflammation, granuloma formation, and interference with vocal fold vibration due to viscoelastic mismatch [11]–[13].

Calcium hydroxylapatite (CaHA) is the only injection material approved by the Food and Drug Administration for vocal fold augmentation. Short- and long-term reviews have demonstrated promising clinical results with minimal complications. [14], [15] For these reasons, CaHA has been suggested for use as a relatively long-lasting material with little concern regarding inflammatory complications, such as those reported with Teflon. [15] Nevertheless, several studies have reported patients who had complications after CaHA injection, including severe inflammation, migration, granuloma formation, and decreased vocal fold vibration. [16], [17] These reports suggest that the risks associated with CaHA may be greater than generally recognized.

With recent advances in biomaterials and tissue engineering, more treatment options are available. Polycaprolactone (PCL) has attracted considerable attention as an optimal biomaterial from many respects. PCL degrades slowly in biological environments, with an *in vivo* degradation time of approximately 2 years. [18] PCL is nontoxic and tissue-compatible, and can eventually be resorbed by the vital organs without abnormal responses. It also exhibits better viscoelasticity or flexibility at room temperature and at 37°C than other conventionally used polymeric biomaterials in clinical practice, such as poly-l-lactide (PLLA) and polyglycolide (PGA). [19] This potentially avoids the problem of decreased vibration of the vocal fold when CaHA is injected in the superficial portion.

Pluronic F127 has been used as a carrier for the homogeneous injection of microspheres because it can reduce their migration. This polymer undergoes a temperature-dependent sol-gel transition. As its lower critical solution temperature (i.e., reverse sol-gel transition temperature) is below ∼24°C, it exists in a gel state at the room temperature and in the body at 37°C. [20] Therefore, the microspheres can be uniformly mixed with the cold Pluronic F127 matrix (sol state), and evenly injected through syringe needle together with the gel state Pluronic F127 at room temperature. Moreover, the gel state Pluronic F127 matrix can reduce the migration of the injected microspheres in the body.

In the present study, we developed a new injection material, consisting of PCL microspheres and Pluronic F127 carrier, for glottal augmentation. The material was injected into rabbits with glottal insufficiency. Endoscopic evaluation was performed every month for 1 year, and histological and radiological analyses were conducted at 1 year after injection. Furthermore, we analyzed the function of the larynx using a high-speed camera system to evaluate its potential for functional vocal fold augmentation *in vivo*.

## Materials and Methods

### Glottal Insufficiency Animal Model

New Zealand white rabbits weighing 2.6 to 3.0 kg were obtained from a laboratory animal company (Koa Tech, Pyeongtaek, Korea) for this study. Experiments were carried out in accordance with the guidelines of the Animal Research Committee, Dongguk University Ilsan Hospital. The protocol was approved by the Institutional Review Board of the Dongguk University Ilsan Hospital (Permit Number: 2011-0154). To make the glottal insufficiency model, the recurrent laryngeal nerve was resected on the left side. Briefly, rabbits were anesthetized with a combination of zoletile 50 (50 mg/kg) and xylazine (4.5 mg/kg) administered intramuscularly. The skin was incised vertically at the midline of the neck. The subcutaneous fat and the strap muscles were separated in the midline until the thyroid gland and tracheal cartilage were encountered. The inferior thyroid vessels were identified at the tracheoesophageal groove, and the recurrent laryngeal nerve was identified by further dissection parallel to the inferior thyroid vessels. The left recurrent laryngeal nerve was cut to a length of 2 cm to prevent spontaneous anastomosis. The subcutaneous tissue was sutured with Vicryl 4-0, and the skin was sutured with Nylon 4-0. All animals were monitored daily, with specific attention to their weight, cough, sputum production, wheezing, and dyspnea.

### Fabrication of PCL Microspheres

**Figure 1:**
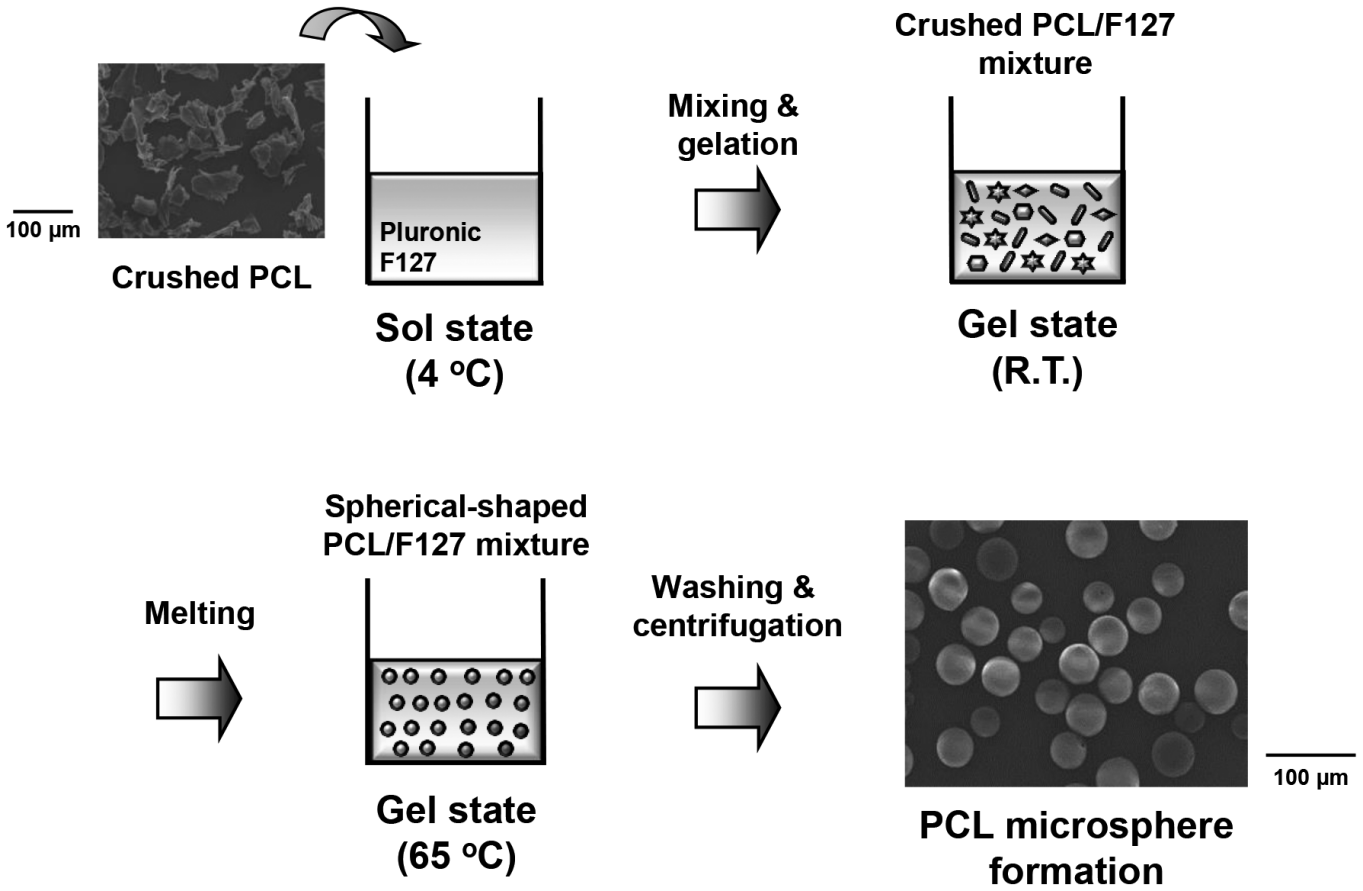
Schematic showing the fabrication process of PCL microspheres by an isolated particle-melting method. The crushed PCL microparticles isolated in a Pluronic F127 gel matrix are melted at a temperature above the melting temperature of PCL, and the molten microparticles are spontaneously constrained into spherical shapes.

PCL (Mw 42500 Da; Aldrich, Milwaukee, WI) and Pluronic F127 (Mw 12500 Da; BASF Corp., Florham Park, NJ) were used to fabricate PCL microspheres. All other chemicals were of analytical grade and were used as received. Water was purified using a Milli-Q purification system (>18 mΩ; Millipore Co., Billerica, MA). For the animal study, the PCL microspheres were sterilized with ethylene oxide, and the Pluronic F127 solution used as a carrier for the homogeneous injection of PCL microspheres was autoclaved. PCL microspheres were fabricated by the isolated particle-melting method described elsewhere. [21] Briefly, PCL pellets were crushed into randomly shaped microparticles using a freezer mill (SPEX 6750; SPEX CertiPrep, Metuchen, NJ). The crushed PCL microparticles were separated using standard testing sieves (Chunggye Industrial Co., Seoul, Korea), and then the microparticles (size, 25–50 µm) were evenly dispersed in 20 wt% Pluronic F127 aqueous solution [PCL microparticles/Pluronic F127 solution, 1/50 (w/v)]. The solution was transformed to the gel state at ∼25°C for 1 h (sol-gel transition temperature, ∼20°C). The PCL microparticles dispersed in the gel matrix were then stored in a water bath at 65°C for 30 min. During this step, the randomly shaped PCL microparticles melted (melting point of PCL, ∼60°C) and were transformed into spheres in the gel matrix. The PCL microsphere/Pluronic F127 gel mixture was rinsed with excess water for 24 h at ∼4°C to remove the Pluronic F127. The PCL microspheres were collected by centrifugation and dried in a vacuum oven overnight (Fig. 1). The morphologies of the PCL crushed microparticles and PCL microspheres were observed by scanning electron microscopy (SEM; Model S-3000N; Hitachi, Tokyo, Japan).

### Injection of PCL Microspheres and Calcium Hydroxylapatite into the Paralyzed Vocal Fold

One week after recurrent laryngeal nerve section, rabbits were anesthetized in the same way. The PCL microspheres were carefully injected into the paralyzed vocal fold of the rabbits (n = 5). Each injection (150 µL/rabbit) was given through a 20 G spinal needle under the guidance of a 4.0-mm, 30° rigid endoscope (Richards, Knittlingen, Germany). To achieve even delivery of the PCL microspheres into the target region, they were mixed with a cold Pluronic F127 aqueous solution (25 wt%; sol-gel transition temperature, ∼17°C) with a microsphere-to-solution ratio of 1/12 (w/v). The PCL microspheres in the Pluronic F127 solution showed a well-packed structure, [21] which would allow the microspheres to maintain a constant volume even after the clearance of Pluronic F127 from the body. The PCL microspheres in the Pluronic F127 gel at room temperature were evenly injected into the paralyzed vocal fold through the syringe needle without separation of the microspheres from the Pluronic F127 gel. The injection procedure was repeated at five rabbits for delivery of the same volume (150 µL) of CaHA (Radiesse®; BioForm Medical Inc., Everdenberg, The Netherlands). Postoperatively, the animals were observed for 2 h before being returned to their cages, where water and standard feed were available. For the following 3 days, the rabbits were given 20 mg/kg kanamycin as prophylaxis. Clinical signs were monitored daily, with special attention to weight, cough, sputum production, wheezing, and dyspnea.

### Laryngoscopic Evaluation of Injected Vocal Folds

After the injection, laryngeal endoscopic examination was performed monthly for 1 year, using a 4.0-mm, 30° rigid endoscope (Richards). Laryngeal images were taken using a digital camera (E4500; Nikon, Tokyo, Japan) attached to the rigid endoscope.

### Radiological Evaluation by microCT and Image Reconstruction

A micro-computed tomography (µCT) scanner (NFR Polaris-G90; NanoFocusRay, Jeonju, Korea) was used to identify the injected material in the larynx 1 year after the operation. Six hundred µCT images were taken at settings of 65 kv, 60 µA, and 500 ms with a resolution of 512×512 in Digital Imaging and Communication in Medicine (DICOM) format. Three-dimensional images of each rabbit’s larynx were reconstructed using software (Lucion; Infinitt Healthcare, Seoul, Korea).

### Recording of Induced Vocal Fold Vibration with High-speed Camera and Vocal Fold Gap Analysis

**Figure 2:**
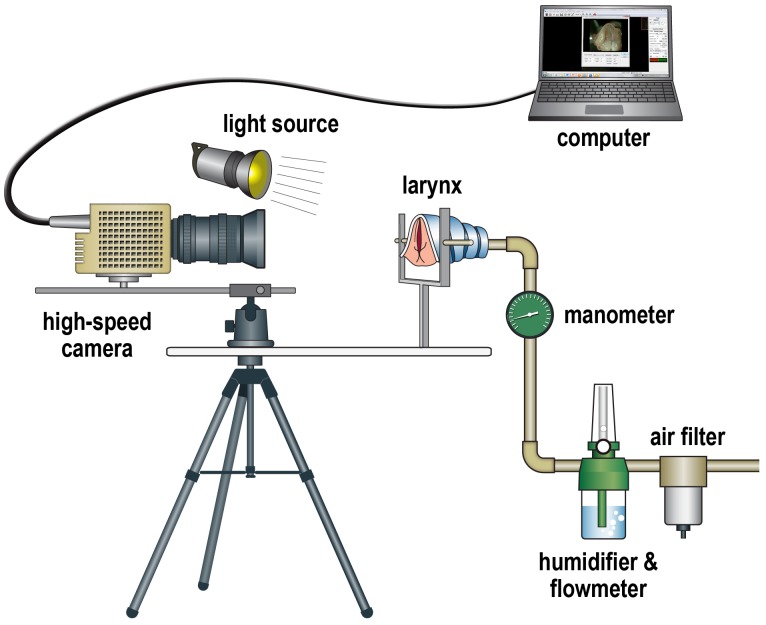
Schematic showing the induced phonation setup for the excised larynx. The excised larynx was fixed with a micrometer, and the vocal fold was vibrated by intratracheal humidified air. The vibrations of the vocal fold were recorded by a high-speed camera, and the data were transferred to a laptop computer.

To perform functional analysis, vocal fold vibrations were examined using an excised laryngeal setup (Fig. 2). After total laryngectomy, the supraglottic structures, including the epiglottis, false vocal folds, and aryepiglottic folds, were removed for better visualization of the true vocal fold. The vocal processes of the arytenoid cartilages were sutured together using Prolene 6-0 to close the glottis. A small segment of the trachea inferior to the larynx was clamped to a pipe using a hose clamp, and the larynx was mounted on a holder. Air, which was filtered (AF20-02; SMC, Shenzhen, China) and humidified with an oxygen flow meter and humidifier (AR20P; Chiyoda Seiki, Yokohama, Japan), was pumped through the pipe to generate the vocal fold vibration. The air pressure was monitored with a manometer (MS-15; Chiyoda Seiki, Yokohama, Japan).

Recordings of the vocal fold vibrations during induced phonation were accomplished by a MotionXtra NR4S2 high-speed video camera (DEL Imaging Systems, Cheshire, CT). A C-mount lens adapter was placed between the image sensor and the macro lens (AF 90 mm F/2.8 Di 1∶1 Macro; Tamron, Saitama, Japan). High-speed video data were recorded at 8000 images per second and a spatial resolution of 256 horizontal×512 vertical pixels. Illumination was provided by a 300-W xenon light source (PS-NP1; Polarion, Seoul, Korea). Each high-speed video data segment consisted of 8000 images.

One cycle of vocal fold vibration consisted of about 10 serial images. From five cycles of vocal fold vibration, the five images with maximal glottal gap area within each cycle were chosen, and the glottal gap area was measured using Image J software (version 1.42; National Institutes of Health, Bethesda, MD, USA, Fig. S1). The glottal gap area was compared between the PCL and CaHA groups by the Mann–Whitney test, using SPSS 21.0 (SPSS Inc., Chicago, IL). P values of less than 0.05 were considered statistically significant.

### Histology of the Larynx and Volume Calculation of the Remaining Material

Animals were humanely sacrificed 1 year after the injection. Specimens from the larynx were fixed in 10% neutral formalin solution, embedded in paraffin, cut into serial sections at a thickness of 4 µm in the axial plane. One in 25 consecutive slides, representing 100- µm intervals in the axial plane, was stained with hematoxylin and eosin (H&E) for light microscopy. All histological slides were reviewed by one pathologist, who was blind to the types of materials, with specific attention to the degree of foreign body reaction around the microspheres at the vocal fold. The volume of the remaining material was calculated by a modification of a previously described method. [22] The dimensions of the remaining injected material were measured in pixels from the stained slides using a microscopic computerized image analysis program (Leica Qwin V3; Konvision Corp., Seoul, South Korea). The volume of the remaining injected material was estimated using the following formula:
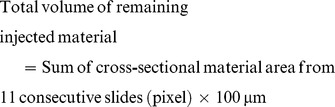



Results for the PCL and CaHA groups were compared with the Mann–Whitney test, using SPSS 21.0. P values of less than 0.05 were considered statistically significant.

### Long-term Toxicity Evaluation

Animals were humanely sacrificed 1 year after the injection. Specimens from the lung, heart, liver, spleen, kidney, and testis were fixed in 10% neutral formalin solution, embedded in paraffin, cut into serial sections at a thickness of 4 µm in the axial plane, and stained with H&E for light microscopy. All histological slides were reviewed by one pathologist, who was blind to the types of materials, with specific attention to toxic reactions in all organs. Blood was sampled from the auricular vein of all rabbits, and the levels of white blood cells, red blood cells, hemoglobin, platelets, total protein, albumin, total bilirubin, glucose, blood urea nitrogen, creatinine, aspartate aminotransferase, alanine aminotransferase, lactate dehydrogenase, calcium, phosphorus, and alkaline phosphatase were determined. Normal values were taken from Laboratory Animal Medicine second edition [23].

## Results

### Fabrication of PCL Microspheres

Figure 1 shows the morphological change of randomly shaped PCL microparticles into spherical particles through the isolated particle-melting method. In this method, crushed (randomly shaped) PCL microparticles isolated in a Pluronic F127 gel matrix are melted by heating above the melting temperature of PCL, and then the molten PCL microparticles are spontaneously constrained into spheres by interfacial forces in the gel matrix, to minimize their surface area (surface/volume ratio) [24].

### Laryngoscopic Examination of the Vocal Folds

**Figure 3:**
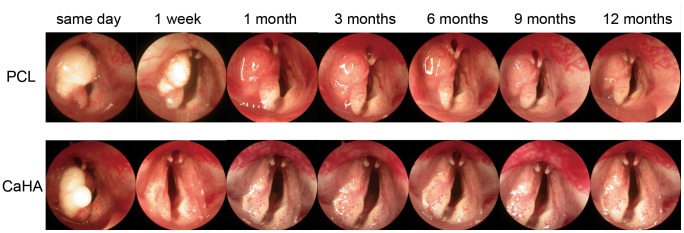
Serial endoscopic images of a larynx injected with PCL microspheres/Pluronic F127 or CaHA were taken for 1 year. From left to right, images were taken immediately after injection, 1-up period. No inflammatory reaction was observed in either group.

Immediately after the injection, PCL microspheres/Pluronic F127 gel remained at the injection site (Fig. 3 upper row), whereas some CaHA leaked out (Fig. 3 lower row). The injected material in the PCL group appeared to remain at the injection site without a significant decrease after 1 year. In contrast, there was a significant reduction of CaHA after 1 week. Markedly more residual material was seen in the PCL group (Fig. 3 upper row) than in the CaHA group (Fig. 3 lower row) throughout the follow-up period. Inflammatory responses such as hyperemia or granulation formation were not identified in any group.

### Radiological Findings from Reconstructed CT Images

**Figure 4:**
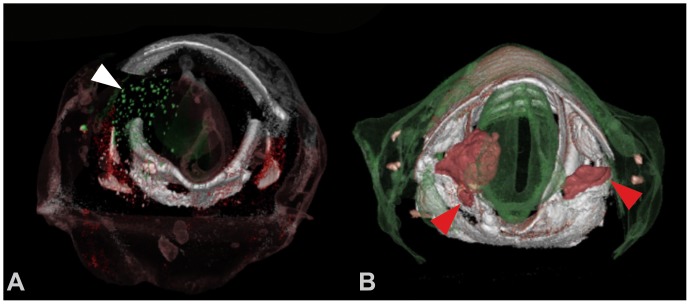
Three-dimensionally reconstructed microCT images of a larynx injected with PCL (A) or CaHA (B). PCL spheres (green dots, white arrowhead) remained at the left vocal fold. CaHA (red arrowheads) migrated to the right vocal fold.

On reconstructed axial CT images, PCL (green dots in Fig. 4A, white arrowhead) remained at the injection site, and CaHA (red portion in Fig. 4B, red arrowheads) was identified at both vocal folds.

### Histological Analyses of the Larynx

**Figure 5:**
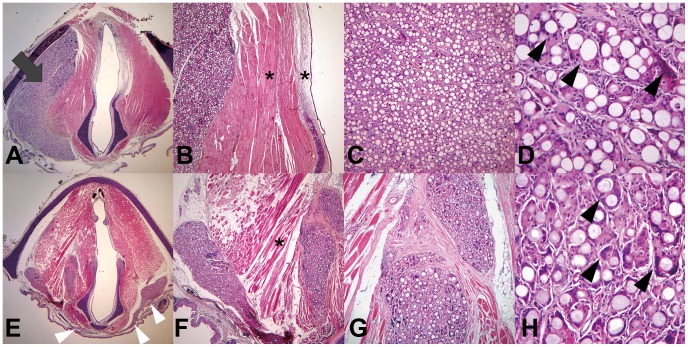
H&E staining of axial cross-sections of the larynx 12 months after injection with PCL microspheres (A–D) or CaHA (E–H). Injected PCL microspheres remained at the left vocal fold (A, ×12.5, gray arrow). In contrast, the CaHA migrated from the left vocal fold, through the fascia space, and to the right vocal fold (E, ×12.5, white arrowheads). Injected PCL (B) and CaHA (F) microspheres did not cause an inflammatory reaction in the surrounding epithelium, lamina propria, or muscle (×40, asterisks). Giant cell formation occurred to the same extent in the PCL (D, ×400, black arrowheads) and CaHA groups (H, ×400, black arrowheads).

**Figure 6:**
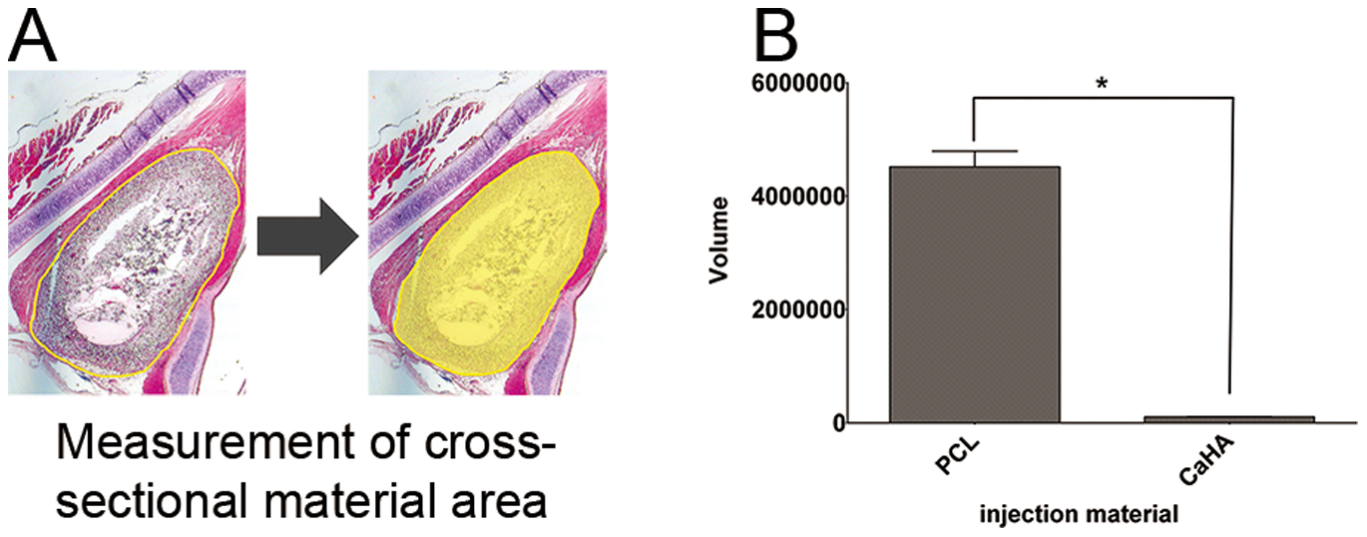
Analysis of residual volume of the injected materials. (A) Method to calculate the cross-sectional material area. (B) Comparison of the remaining volumes of injected PCL and CaHA 12 months after injection. *: P<0.01.

Injected PCL microspheres remained at the injection site (Fig. 5A, gray arrow), whereas CaHA migrated to the contralateral vocal fold (white arrowheads in Fig. 5E) through the potential fascia space at the posterior portion of the larynx (Fig. S1). Neither material caused an inflammatory response in the nearby muscle nor lamina propria (asterisks in Fig. 5B, F). The same degree of giant cell formation, which is a normal response to long-lasting material, was found in both groups (black arrowheads in Fig. 5D, H). The volume of residual injection material was significantly larger in the PCL group (Fig. 6, *P* = 0.009).

### Vocal Gap Closure Analysis from Images taken by the High-speed Camera

**Figure 7:**
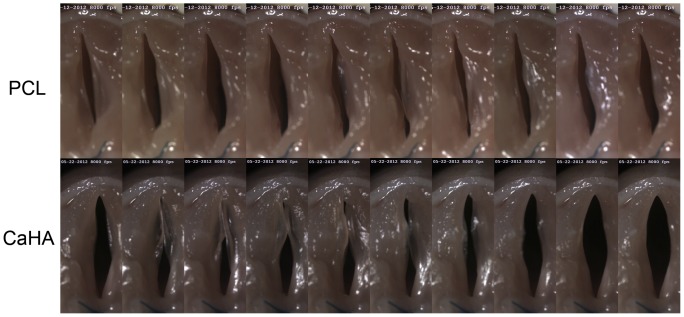
Serial vocal fold vibration images taken by a high-speed camera at a speed of 8000 frames per second. The vocal fold gap was wider in the CaHA group than in the PCL group.

**Figure 8:**
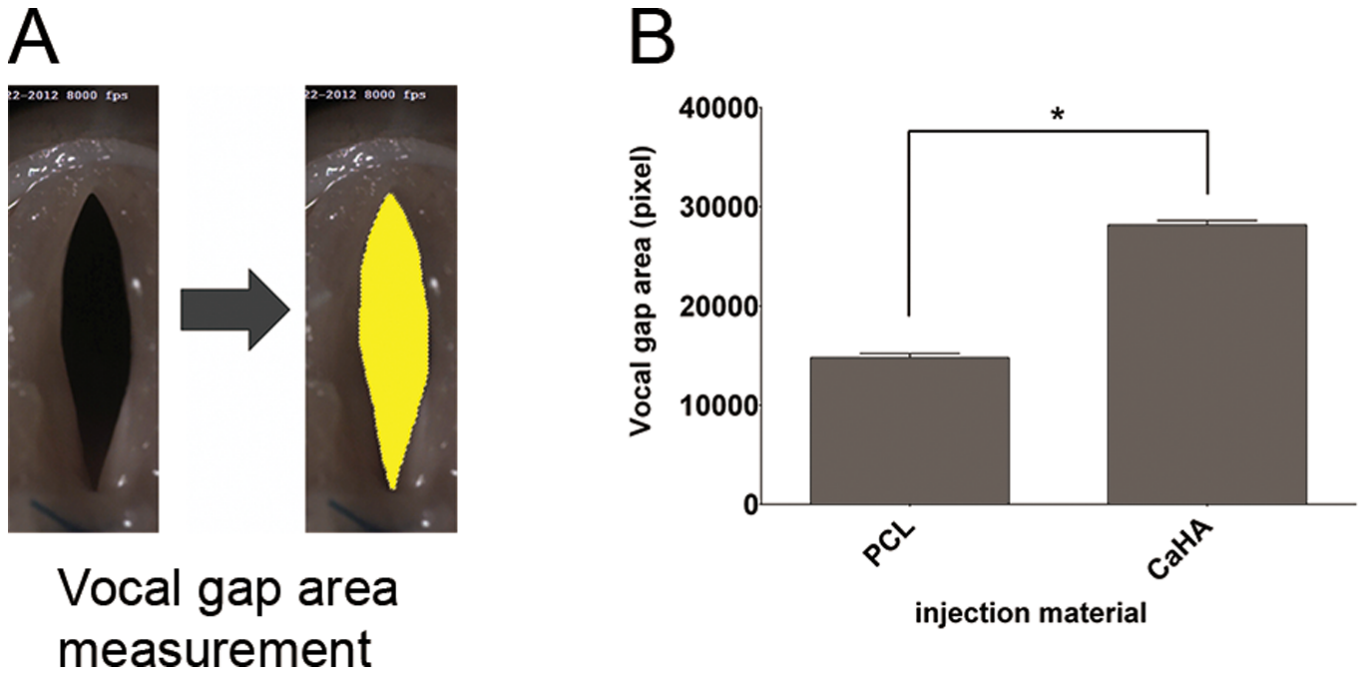
Analysis of vocal fold gap area from images with maximal glottal gap area within each cycle of vocal fold vibration. (A) Method to calculate the vocal gap. (B) Comparison of the vocal gap area measured 12 months after injection of PCL or CaHA. *: P<0.01.

Serial photographs during one cycle of vocal fold vibration showed a much larger gap between the two vocal folds in the CaHA group (Fig. 7) than in the PCL group. The vocal gap area was significantly smaller in the PCL group (Fig. 8, *P* = 0.008).

### Long-term Toxicity Evaluation

From the histological slides, no toxic reactions were identified in any other organ (Fig. S2). All blood tests, except glucose, were within normal limits. All animals showed high glucose levels, and there were no significant differences in any parameters between the groups.

## Discussion

Optimal vocal function requires an aerodynamically competent glottal valve and pliable phonatory mucosa. Over the past century, surgeons have attempted to repair aerodynamic incompetence by augmenting the vocal folds. Several materials have been tested by injection at the paralytic vocal fold to restore glottal closure. However, there have been no reports of a reliable injection material to restore normal suppleness to paralyzed vocal folds. Though the fat is autologous, may be generously injected, and is readily available, it is typically harvested in the operating suite under sterile conditions. Another problem of autologous fat injection laryngoplasty is whether the injected fat maintains the graft volume. The fate of injected fat continues to be debated. The survival is highly variable, which may be due to fat preparation techniques; indeed, many suggest substantial overinjection of due to this immediate variability. [22], [25] Polytetrafluoroethylene, or Teflon, is a permanent injectable that is of considerable historical significance but has fallen out of favor in the past 20 years. Long-term studies have revealed significant foreign-body inflammatory reactions to this substance, often requiring removal with subsequent significant vocal fold tissue loss. Presently, Teflon it is rarely used, with newer substances providing safer alternatives. [11] Bovine-based collagen products have been used for injection laryngoplasty. There is a very small potential for allergic response to this material. Thus, the FDA advocates skin hypersensitivity testing prior to using this substance [26].

This study was performed to evaluate the effects of PCL microspheres/Pluronic F127 hydrogel on vocal fold vibratory patterns in a unilateral vocal fold palsy animal model. In a previous pilot study in animals, we demonstrated excellent tolerance of this bioimplant injected into the paralyzed vocal fold; no adverse reactions were observed over a 12-week follow-up period. [27] We performed the present long-term study to confirm the good results obtained in these first preclinical trials. Overall, a comparative analysis of the histological results over a period of 12 months indicated that in contrast to Radiesse®, PCL microspheres/Pluronic F127 remained more in volume at the paralyzed vocal fold, without inducing inflammation or migration.

In a clinical setting, it is necessary to inject more CaHA than the amount needed for vocal fold contact, as the injection of the precise amount of CaHA that is needed for vocal fold contact results in loss of the effect on glottal contact soon after injection. However, the biodegradation time of CaHA is as long as 1 year. [2] This raises the question of why the effect on glottal contact is lost so soon after injection. The CaHA microspheres range in size from 25 to 45 µm and are suspended in an aqueous gel composed of water, glycerin, and sodium carboxymethylcellulose. Once injected, the body absorbs the gel carrier, and the microspheres are left behind [28] Carboxymethylcellulose (CMC), which is used to deliver the CaHA spheres, accounts for 20% of the injected volume. Therefore, the volume decreases after CMC is absorbed. [29] In addition, CaHA is redistributed in the vocal fold or may even migrate through the puncture wound caused by the injection needle, as demonstrated in the present study. CaHA was reported to pass easily into unplanned locations either during placement or during coughing or phonation postoperatively. [16] This would cause loss of the augmentation effect soon after injection. However, the injection volume of CaHA needed to ensure a sustained effect is not known. The novel PCL microspheres/Pluronic F127 hydrogel material described here has advantages over CaHA. Pluronic F127 can tightly hold the PCL at the injection site. Thus, the volume of residual injection material was significantly larger in the PCL/Pluronic F127 group than CaHA group. It is well-known that the Pluronics gel is rapidly absorbed in the body by dilution from the body fluid [30]–[32]. Oh *et al.* revealed that the Pluronics gel is totally disappeared in PBS (37°C) and peritoneal cavity (rat) at 3 days after application. [33]. Thus, effect of Pluronic was exerted during injection or immediately after the injection. One week after injection, we can consider that most of the augmented volume was by the PCL microspheres.

Furthermore, the PCL microspheres in the Pluronic solution showed a well-packed structure, [21] allowing them to retain a constant volume even after the clearance of Pluronic F127 from the body. Therefore, laryngologists can inject the exact volume of PCL microspheres/F127 hydrogel that is needed for vocal fold augmentation.

Although we injected all materials into the left vocal fold, we identified CaHA in the right vocal fold, raising questions regarding how the CaHA migrated to the contralateral vocal fold. Fascial planes divide the neck into true and potential spaces. The two main fascial divisions of the neck are the superficial cervical fascia and the deep cervical fascia. These further divide into three layers. The fascial layers separate important structures in the neck. In this way, they form anatomical walls against the spread of infections, although this alignment could allow the spread of air, infection, or injectate throughout connected fascial spaces. [34], [35] CaHA injected between fascial planes may migrate to the contralateral vocal fold during coughing, swallowing, or injection [16].

Another advantage of PCL microspheres/Pluronic F127 hydrogel compared with CaHA is that its viscoelasticity is a closer match with that of the vocal fold. Because CaHA is stiff, it can decrease vibration of the vocal fold when injected into the superficial layer. [2] CaHA may affect vibration of the vocal fold even when contained within the muscle layer. [36] The elastic modulus of PCL is about 0.4 GPa, [37] as compared with 100 GPa for CaHA. [38] Therefore, PCL should interfere with vocal fold vibration much less than CaHA, when injected at the same depth.

The results of all blood tests, except glucose, were within normal limits. As the animals lived in cages, the high levels of glucose in all animals might have been due to a lack of exercise.

We used a high-speed camera to visualize vocal fold vibrations. In humans, the actual vocal fundamental frequencies vary by sex, with values of approximately 200 Hz and 120 Hz in females and males, respectively. [39] A high-speed imaging system is essential to provide high frame-rate images for the analysis of this high-frequency movement. High-speed digital imaging overcomes the limitations of videostroboscopy, which is currently the most widely used clinical technique for laryngeal imaging. [40] A well-known drawback of videostroboscopy is its reliance on periodic voice signals. Capturing 30 frames per second (fps), videostroboscopy creates a slow-motion illusion of vocal fold vibration by compiling images at different points of each vibratory cycle. [41] As it creates a composite image averaged over several vibratory cycles, videostroboscopy can only be used to record periodic vocal fold vibrations. Furthermore, because the strobe light and camera are programmed at slightly different frequencies to image successive points in the glottal cycle, the activation of the strobe light relies on a stable acoustic phonation frequency. [42] Another notable drawback of videostroboscopy is that many voice disorders are marked by either aperiodicity or fluctuating frequency, and therefore cannot be visualized with this technique. [43] High-speed digital imaging records a full image of the vocal folds with high temporal resolution, a significant advantage over videostroboscopy. [44] Thus, it allows for quantitative observations of irregular and aperiodic vocal fold vibrations, which often occur in asymmetric vocal folds with incomplete glottal closure. Patterns of asymmetric and aperiodic vocal fold vibrations associated with unilateral vocal fold paralysis have been reported in the literature. [41], [45], [46] Few studies have utilized high-speed digital imaging to quantify the vibration, because of its high cost. [47] A recent study reported that 2000 fps, a typical frame rate used in high-speed imaging, is insufficient when evaluating the mucosal wave characteristics. [48] We used a frame rate of 8000 fps, which to our knowledge is the highest frame rate available in color [40], [49]; thus, we used the most advanced and accurate high-speed camera system available to evaluate vocal fold vibration in the present study.

The major limitation of this study was the absence of acoustic and speech aerodynamic measurements, which are ordinarily conducted with human patients. Post-injection vocal fold vibration was measured using an induced phonation method. Unfortunately, this laboratory technique does not equate with the appraisal of natural voice production behaviors in a live unanesthetized human patient to analyze the functional outcome of laryngeal surgery. However, to our knowledge, no appropriate animal model has yet been developed, as there are no animals with the same vocal fold structure as that of humans or capable of speaking like a human. The present study did not focus on evaluating the phonation characteristics of rabbits. The aim of this study was to evaluate the efficacy of PCL microspheres/Pluronic F127 hydrogel as an injection material for augmentation of vocal fold volume, and this was fulfilled by the methods adopted.

## Conclusions

In conclusion, this long-term study suggested that the novel injection material PCL microspheres/Pluronic F127 hydrogel is better than commercially available CaHA for restoring glottal closure in an animal model of glottal insufficiency. Moreover, PCL microspheres/Pluronic F127 hydrogel exhibits *in vivo* biocompatibility without migration or leakage through the injection site. Therefore, vocal fold augmentation was almost completely retained, without inflammation or granuloma formation, during the 12-month follow-up period of this study.

## Supporting Information

Figure S1 H&E staining of larynx to show the migration pathway (black arrowheads) of CaHA. CaHA migrated to the contralateral vocal fold along the fascia space posterior to the cricoid cartilage (*: cricoid cartilage).
(TIF)

Figure S2 H&E staining of other organs to rule out systemic toxicity of PCL. There was no significant inflammatory response at lung, kidney, liver, heart, spleen, and Kidney.
(TIF)
