# Supplementary figures and images for: Vocal Fold Augmentation with Injectable Polycaprolactone Microspheres/Pluronic F127 Hydrogel: Long-Term In Vivo Study for the Treatment of Glottal Insufficiency
Source: PLoS One. 2014 Jan 22;9(1):e85512. doi: 10.1371/journal.pone.0085512 (PMC3899012; doi:10.1371/journal.pone.0085512)

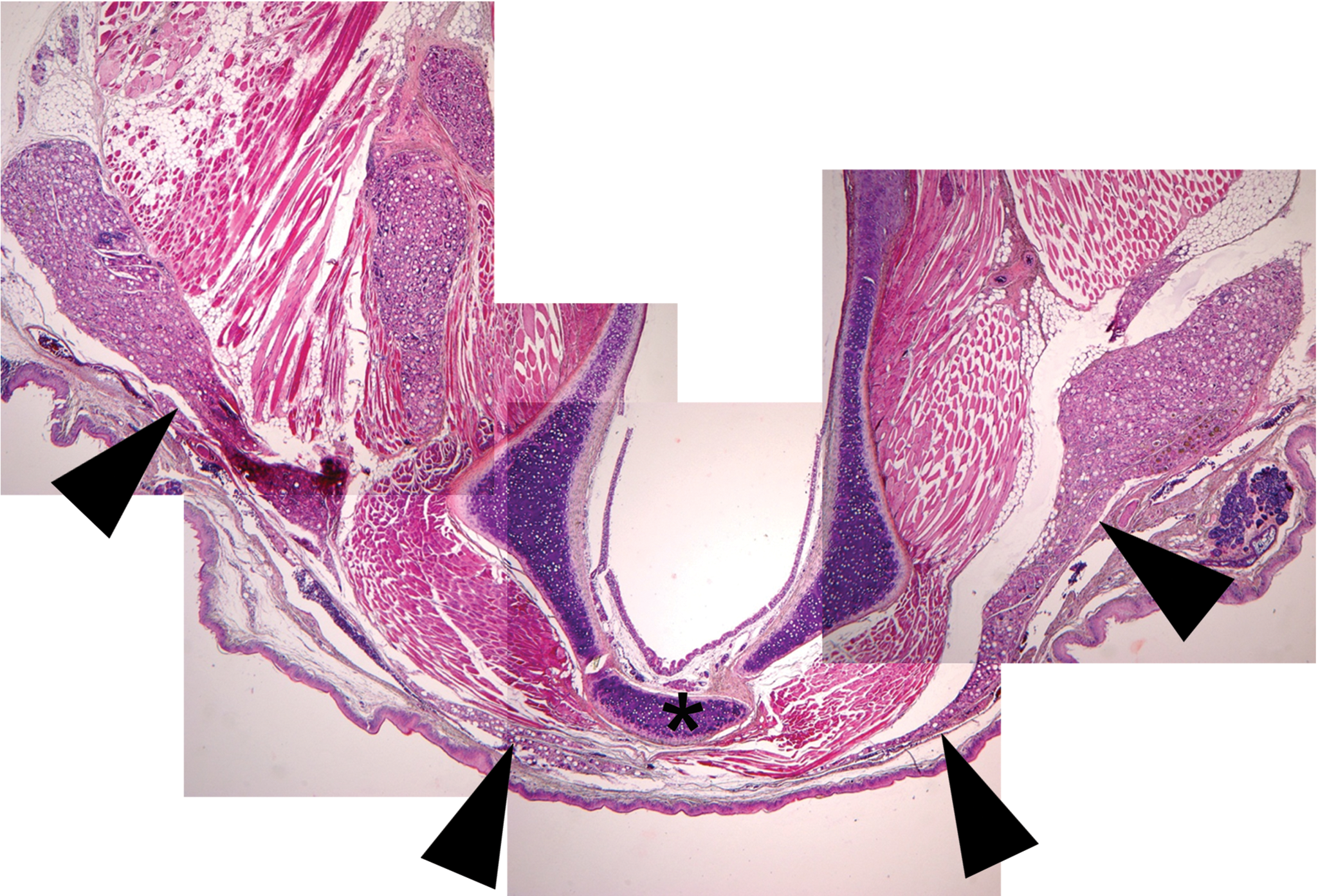

Supplement: Figure S1 — H&E staining of larynx to show the migration pathway (black arrowheads) of CaHA. CaHA migrated to the contralateral vocal fold along the fascia space posterior to the cricoid cartilage (*: cricoid cartilage). (TIF) [file pone.0085512.s001.tif]

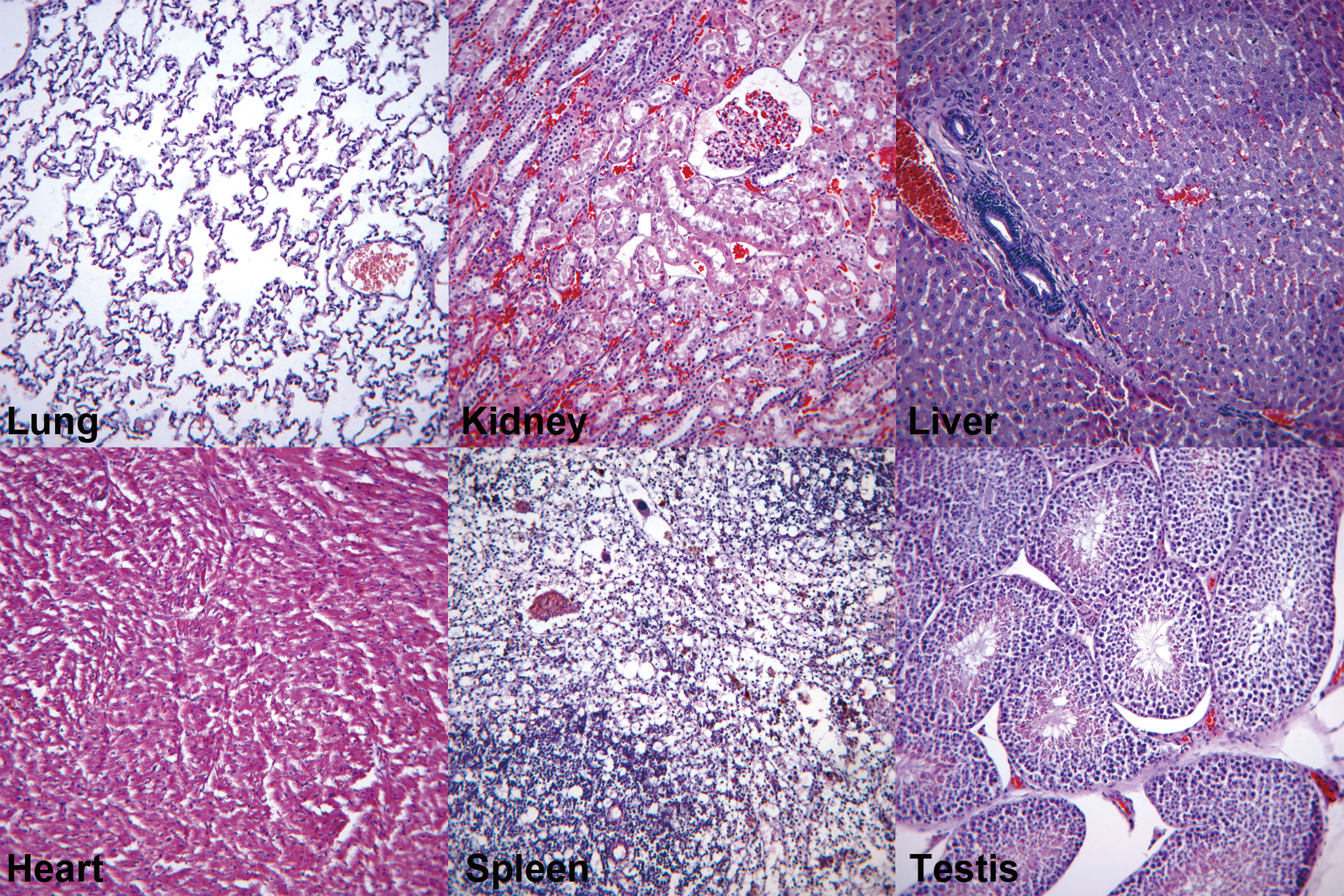

Supplement: Figure S2 — H&E staining of other organs to rule out systemic toxicity of PCL. There was no significant inflammatory response at lung, kidney, liver, heart, spleen, and Kidney. (TIF) [file pone.0085512.s002.tif]
